# Short measures of youth psychopathology: psychometric properties of the brief problem monitor (BPM) and the behavior and feelings survey (BFS) in a Norwegian clinical sample

**DOI:** 10.1186/s40359-022-00894-6

**Published:** 2022-07-24

**Authors:** Kristian Rognstad, Siri Saugstad Helland, Simon-Peter Neumer, Silje Baardstu, John Kjøbli

**Affiliations:** 1 Center for Child and Adolescent Mental Health, Eastern and Southern Norway, Oslo, Norway; 2 Regional Centre for Child and Youth Mental Health and Child Welfare, UIT The Arctic University of Norway, Tromsø, Norway; 3 Department of Child Health and Development, Norwegian Institute of Public Health, Oslo, Norway; 4 Department of Education, University of Oslo, Oslo, Norway

**Keywords:** Brief Problem Monitor, Behavior and Feelings Survey, Psychometrics, Measurement models, Validity of measures

## Abstract

**Background:**

Tracking clinical outcomes during therapy can be useful for improving both clinical practice and research. For repeated data collection, short, reliable, and valid measures of central aspects of psychopathology are necessary. The current paper investigates the psychometric properties of two short surveys for measuring central dimensions of psychopathology in youth.

**Methods:**

We investigated the factor structure and validity of the Norwegian translations of the Behavior and Feelings Survey (BFS) and the Brief Problem Monitor (BPM). The BFS has previously shown a two-factor structure and indications of validity as a measure of internalizing and externalizing problems in youth. The BPM has support for a three-factor structure of internalizing, externalizing, and attention problems. In our sample of 503 patients (56% female, age 6 to 18) in a Norwegian outpatient clinic, we conducted confirmatory factor analyses to test the assumed measurement models and further considered the concurrent validity of the measures.

**Results:**

Internal reliability of both measures were good. The results suggest that the assumed measurement models for both questionnaires only partly fit our data but that subscales of the BFS and BPM still indicate convergent validity. Scores on subscales (internalizing and externalizing problems) on both measures converged with relevant subscales as well as with relevant groups of diagnoses.

**Conclusions:**

Alternative measurement models, and the usefulness and limitations of these short-form questionnaires for internalizing and externalizing problems, are discussed.

## Introduction

Psychotherapy is generally helpful for youth with mental health problems [1], but a significant group does not seem to experience symptom relief [2]. A development within psychotherapy that attempts to address this issue is the increased emphasis on outcome measurement and measurement-based care. Measurement feedback systems (MFS) use self-reports or other data sources to continuously collect information about patients in therapy. Such data can inform treatment decisions made by the therapist throughout the course of therapy. In this sense, patient-reported outcome measures can potentially increase treatment effects as indicated by several reviews [3–5].

Prerequisites for a useful MFS are that the included measures have sound psychometric properties and that data collection can be implemented. When measurement is conducted repeatedly over the span of a therapy period, the number of items is highly relevant. Shorter forms are less taxing for respondents and are likely to increase response rates. Brief surveys are also preferable in research to ensure high response rates and attention to items.

Additionally, feedback should be of clinical relevance and the measures thus gauge concepts that are appropriate for the patient population. On this background, this study aims to evaluate two short measurements for central dimensions of psychopathology.

Dimensional models for the classification of child and adolescent psychopathologies have for a long time been dominant, and multiple models with different levels of specificity have been proposed and gathered empirical support. A model of one general psychopathology dimension, *p*, can be supported empirically [6], but more fine-grained models can also show predictive capacities of more narrowband dimensions [7, 8]. A two-dimensional model for the classification of child and adolescent psychopathologies—internalizing and externalizing problems—is often regarded as a parsimonious and adequately precise way to capture child and adolescent disorders [9, 10]. The internalizing dimension is characterized by negative emotion (e.g. anxiety and depression), while the externalizing dimension is distinguished by problems with inhibition (e.g. substance abuse and conduct disorders) [10]. Thus, reporting on patients’ fluctuation on these dimensions can be useful for treatment and research.

### The brief problem monitor (BPM)

One frequently used set of measures in youth populations is the Achenbach System of Empirically Based Assessment (ASEBA) forms, which include the Child Behavior Checklist (CBCL) and the Youth Self-Report (YSR) [11]. The Brief Problem Monitor Parent (BPM-P) and Youth (BPM-Y) forms are short versions of the CBCL and the YSR, respectively. These shorter forms consist of 19 items and are designed to measure the three factors of internalizing, externalizing, and attention problems [12].

The BPM has been developed through factor analysis and item response theory (IRT) for a large archival CBCL dataset [12]. Different studies have provided evidence that the BPM meets some important psychometric standards. In Piper et al. [13] the BPM-P had good internal consistency for the full scale (Cronbach’s alpha = 0.91) and the subscales (Cronbach’s alpha = 0.78–0.87) and excellent correspondence with both the CBCL total score (R^2^ = 0.90) and its subscales (internalizing R^2^ = 0.74, externalizing R^2^ = 0.86, and attention problems R^2^ = 0.94). The Spanish translation of BPM-P has shown an adequate fit for the expected 3-factor model in a sample of 6 to 8-year-old children, as well as concurrent validity with parent ratings on CBCL and Strength and Difficulties Questionnaire [14]. The Norwegian translation of the BPM has been found to have acceptable reliability and correspondence with the long version of ASEBA to indicate validity [15]. In a more recent study with a national Norwegian sample, BPM-P had an excellent model fit for the three-factor model in a sample of 8–12-year-old children identified as being at risk for internalizing problems [16].

### The behavior and feelings survey (BFS)

The Behavior and Feelings Survey (BFS) is a 12-item survey that measures internalizing and externalizing problems in children and adolescents [17]. The BFS is very brief and thus minimizes the measurement burden. In contrast to the BPM, the BFS is freely available (https://weiszlab.fas.harvard.edu/measures), which eliminates financial and copyright barriers.

The BFS was developed and evaluated in four samples of youths, and the original English version has demonstrated a robust factor structure, good internal consistency, test–retest reliability, and convergent and discriminant validity [17]. The scale was developed in several steps: (1) youth and caregivers generated “top problems” and, based on these, an expert panel selected 48 items; (2) youths and caregivers answered the survey, and the number of items was reduced using IRT; (3) two latent factors were identified through exploratory factor analysis and each factor was reduced to 6 items using IRT; (4) the validity of 12-items BFS version was tested in a new sample; and (5) its sensitivity to change was tested to consider its performance as a progress monitoring tool. In Weisz et al. [17], the BFS met accepted psychometric standards and was shown to be sensitive to change during treatment. It had good internal consistency for both caregiver and youth reports for the total score (respectively α = 0.87, and α = 0.87) and for both the internalizing (α = 0.84, and α = 0.91) and the externalizing (α = 0.94, and α = 0.89) scales. Associations between BFS total and CBCL/YSR total scores were high for both caregiver (r = 0.61, *p* < 0.001) and youth (r = 0.72, *p* < 0.001) reports, indicating convergent validity.

Both BPM and BFS measurement systems include youth and caregiver versions, which is important as otherwise reliable measures have revealed modest cross-informant correlations, indicating that multiple perspectives are necessary [18–20].

### Associations between scales and diagnoses

Some categories of the International Classification of Diseases (ICD) [21] contain mainly internalizing or externalizing symptoms. Measures of internalizing and externalizing problems should thus have some relation to the diagnoses young people are given. Some studies have found links between groups of patients with different diagnoses and their scores on ASEBA CBCL/YSR and BPM. Associations have been established between the internalizing scale of ASEBA (CBCL/YSR) and general diagnostic groups (e.g. anxiety and affective disorders) as well as with more specific diagnostic groups (e.g., conduct disorder) [22]. In Ebesutani et al. [23], CBCL scales corresponded with related clinical diagnoses, although the prediction accuracy varied from not significantly better than chance (for the Aggressive behavior scale) to fair (e.g., Withdrawn/Depressed scale) or good (e.g., Anxiety/Depressed scale). Similarly, the BPM-P internalizing score has proved useful in identifying service use for mental health problems as well as impaired functioning among 6- to 8-year-old children [14]. Piper et al. [13] reported that patients with a depression diagnosis had scores on the BPM internalizing scale that were 3.6 times higher than those reporting no diagnosis, and patients with an anxiety diagnosis had BPM internalizing scores 3.2 times higher. To our knowledge, our study is the first study to tie BFS scores to patient diagnoses.

In the current study, we were interested in whether the BPM and BFS could remain reliable and valid in Norwegian and in a Norwegian specialist health service context. The purpose of the study was to (1) test the BFS and BPM factor structure, (2) examine the reliability, and (3) evaluate the convergent validity of the translated BFS and BPM when used in a youth outpatient population in Norway.

## Methods

### Participants

503 patients were recruited from an outpatient clinic in Norway. Respondents were both patients (n = 386) and caregivers (n = 412). Patients came to therapy either by themselves (often adolescents), with their caregiver, or were represented only by caregivers receiving parental guidance (usually the youngest patients). This resulted in a difference in the number of responses from patients and caregivers as respondents are the person(s) that met at the clinic for the first session and were able to fill out the pre-treatment assessment. The patients were between 6 and 18 years old (mean age = 12.73, SD = 3.15) and 56% female. The clinic is part of the Norwegian specialist health service where patients should have one or more diagnoses registered, and in our sample, the most frequent were anxiety disorders (35%), depression and mood disorders (22%), and behavioral and attention disorders (22%).

### Measures

The Brief Problem Monitor has parallel forms for self-report for youth (BPM-Y), caregiver reports (BPM-P), and teacher reports (BPM-T). Each item is rated on a scale from 0 (not true) to 2 (very true). The 19-item form is designed to measure three factors: seven items measuring externalizing problems (e.g., “I argue a lot” and “I disobey my parents”), six items measuring internalizing problems (e.g., “I feel worthless or inferior” and “I am too fearful and anxious”) and six items measuring attention difficulties (e.g., “I fail to finish things I start” and “I have trouble sitting still”).

The Behavior and Feelings Survey (BFS) consists of 12 items. Each item is rated from 0 (not a problem) to 4 (a very big problem) over the past week. Six items are intended to measure internalizing problems (e.g., “I feel sad” and “I worry about bad things happening”) and six items to measure externalizing problems (e.g., “I refuse to do what adults tell me to do” and “I argue with people"). The BFS has parallel self-report and caregiver forms.

The diagnoses followed the diagnostic system of the ICD 10th Revision (ICD-10). Multiple diagnoses could be given and in the current dataset, patients had up to four diagnoses. The research team gave no instructions for the process of diagnosis and diagnoses in the dataset were extracted from patient journals after therapists followed ordinary procedures at the clinic.

### Procedure

The data collection and processing were approved by the Regional Committee for Medical and Health Research Ethics—South East and further guidance for consent, privacy, and data protection was given by the Norwegian Center for Research Data. Patients were informed about the study through a letter before their first session. Upon arrival for the session, they were given a tablet with study information and, if consenting, filled out questionnaires on the tablet. Participants were instructed to fill out the forms independently, but younger patients could have items read or explained to them if necessary.

The Norwegian versions of both the BFS and BPM used were translated by Norwegian expert committees of researchers and psychologists and back-translated and reviewed by the original developers in accordance with WHO recommendations for the translation and adaptation of instruments [24].

### Analytic strategy

Reliability scores, correlation calculations, and linear regressions were performed using IBM SPSS Statistics v. 27 [25]. Confirmatory factor analyses were conducted using Mplus 8.6. [26].

#### Reliability

For both BPM and BFS, Cronbach’s alphas were used to measure internal consistency for subscales and total score, for both caregiver and youth versions. Internal consistency can be considered excellent when Cronbach’s alpha is ≥ 0.80, good when 0.70 ≤ r < 0.80, adequate when 0.60 ≤ r < 0.70 and inadequate when < 0.60 [27].

As a measure of cross-informant agreement, intraclass coefficients (ICC; two-way random model with measures of consistency) were calculated to assess agreement across youth and caregiver reports. In line with Koo & Li [28], we based our evaluation of the ICC values on a 95% confidence interval of the ICC estimates and considered values < 0.5 as poor, 0.5–0.75 as moderate, 0.75–0.90 as good, and > 0.90 as excellent.

#### Confirmatory factor analysis

We performed confirmatory factor analyses (CFA) to examine the BPM’s intended three-factor structure of internalizing, externalizing, and attention problems [12]. Informed by the exploratory factor analysis (EFA) in Weisz et al. [17], we conducted a CFA of the BFS to consider whether the same factor structure was reproduced in our sample using the Norwegian translation. All CFAs were conducted for youth and caregiver data separately.

Variables were treated as categorical as items were not assumed to have the same interval between each response alternative. The data from BFS and BPM total scores also failed tests of normality (both Kolmogorov–Smirnov and Shapiro–Wilk tests). Thus, a weighted least square mean and variance adjusted (WLSMV) estimation was applied [29]. Factor loadings were examined to determine whether they adhered to the assumed model, with substantial loadings on expected factors and no cross-loadings.

Given the large sample size, the chi-square test for model fit is not eligible as this fit index might lead to the rejection of reasonably well-specified models. Thus, several other fit indices were considered, including the root mean square error of approximation (RMSEA), the Tucker-Lewis index (TLI), the comparative fit index (CFI) and the Standardized Root Mean Squared Residual (SRMR). CFI and TLI values of > 0.95 indicate an acceptable model fit [30], RMSEA < 0.08 indicates a fair fit [31], and the approximate fit is defensible with SRMR ≤ 0.08 [32]. Modification indices were consulted in cases of poor model fit.

#### Tests of validity

Convergent validity is an estimate of the measure’s ability to agree with the outcome for another measure intended to measure the same construct. Concurrent convergent validity is good when test scores have a strong relationship with a measurement of the same construct administered at the same time or shortly after. In the current study, we assessed convergent validity by exploring the extent to which scores on the BFS are correlated with scores from the BPM. Pearson product-moment correlation coefficients were calculated for each of the BFS and BPM subscales’ covariance with each other.

In addition, we considered whether patients with internalizing or externalizing diagnoses scored higher on internalizing or externalizing in the BFS/BPM subscales. Most participants in our sample had been given one or more diagnoses from ICD-10. Although the ICD-10 does not use the internalization/externalization dimensions as a theoretical framework, there should be some relation between scores on the subscales and a young person's diagnoses. Diagnoses were dummy coded for the presence of internalizing diagnosis and presence of externalizing diagnosis (see appendix for an overview of categorization of diagnoses). We compared the scores on relevant subscales for patients with and without internalizing and externalizing diagnoses separately.

## Results

### Descriptive statistics

Mean scores and standard deviations for the BFS and BPM total scale and subscales are presented in Appendix 1. Girls reported significantly higher levels of problems than boys (*p* < 0.01) in both BFS and BPM. The difference emerged from higher scores on internalizing subscales. In caregiver reports, girls scored higher than boys on internalizing problems and boys scored higher than girls on externalizing problems, in both BFS and BPM.

ASEBA provides separate BPM norm scores for boys and girls, one set for 6–11-year-olds and one set for 12–18-year-olds. For the BPM-Y and BPM-P, we calculated T scores separately for participants 6–11 and 12–18 years old and for boys and girls by plotting the average scores from each subgroup in our sample into the BPM/6–18 software [32]. For both BPM-Y and BPM-P, Norway is among the societies in BPMs multicultural norm group 1 (relatively low problem scores) and T scores were computed accordingly by choosing this option in the scoring software. While T scores < 65 are considered to be in the normal range, scores of > 65 are regarded as elevated and to warrant concern [12]. For participants of all age groups and both genders, the average T scores for the overall BPM-P were in the elevated range (T scores = 66–67). BPM-Y among the 11–18-year-old was less elevated (boys overall T score = 60, girls overall T score = 65). The internalizing subscale was elevated in all groups and respondents (T scores = 67–69), except in the self-report from boys (T score = 63). T scores for other subscales were more mixed (externalizing range = 52–65, attention problems range = 59–63).

### Tests of reliability

Internal reliability was good for the BFS youth (Cronbach’s α = 0.84), and for subscales of internalizing (Cronbach’s α = 0.89) and externalizing problems (Cronbach’s α = 0.84). For the BFS caregiver version, internal reliability was equally good (Cronbach’s α = 0.85), and excellent for subscales of internalizing (Cronbach’s α = 0.90) and externalizing (Cronbach’s α = 0.91) problems. Internal reliability was good for both the BPM-P (total Cronbach’s α = 0.84; internalizing Cronbach’s α = 0.81; externalizing Cronbach’s α = 0.85; attention problems Cronbach’s α = 0.84) and the BPM-Y (total Cronbach’s α = 0.85; internalizing Cronbach’s α = 0.84; externalizing Cronbach’s α = 0.74; attention problems Cronbach’s α = 0.78).

Cross-informant agreement, between patient and caregiver reports, was indicated by an intraclass correlation coefficient of 0.64 (*p* < 0.001) for the BFS total score, 0.73 (*p* = 0.001) for the BFS internalizing subscale, and 0.69 (*p* < 0.001) for the BFS externalizing subscale. For the BPM, the agreement was lower, with an intraclass correlation coefficients of 0.52 (*p* < 0.001) for the BPM total, 0.65 (*p* < 0.001) for the internalizing scale, 0.67 (*p* < 0.001) for the externalizing scale, and 0.64 (*p* < 0.001) for the attention problems scale.

### Factor structure

#### BPM factor structure

The three-factor model previously shown for the BPM was not confirmed in the current sample for neither the caregiver nor the youth report. For the BPM-P, none of the indices for approximate model fit were acceptable (RMSEA = 0.10, TLI = 0.88, CFI = 0.90, SRMR = 0.11). BPM-Y was only on par with two of the four model fit indices in our analysis plan (RMSEA = 0.06 and SRMR = 0.08, but with CFI = 0.94 and TLI = 0.93). Introducing a new factor by splitting internalizing into depression and anxiety items significantly increased model fit. This resulted in a four-factor model that performed well on all four investigated model fit indices for youth reports (RMSEA = 0.05, CFI = 0.96, TLI = 0.95, SRMR = 0.07); however, model fit was still inadequate for the caregiver report data (RMSEA = 0.10, CFI = 0.91, TLI = 0.89, SRMR = 0.10).

In this four-factor model, four items loaded on depression (in BPM-Y 0.67–0.83, in BPM-P 0.61–0.77), two items loaded on anxiety (in BPM-Y 0.87–0.88, in BPM-P 0.80–0.95), seven loaded on externalizing (in BPM-Y 0.50–0.79, in BPM-P 0.64–0.87) and six loaded on attention problems (in BPM-Y 0.50–0.89, in BPM-P 0.53–0.92).

As BPM-Y is intended for use in youth from 11 to 18 years old, we did a similar CFA including respondents from this age group only (n = 290). The model fit for the three-factor model did not reach acceptable levels on the investigated indices (RMSEA = 0.06, CFI = 0.94, TLI = 0.94, SRMR = 0.09). The described four-factor model had adequate model fit on all indices for BPM-Y answered by youth 11–18 years old (RMSEA = 0.05, CFI = 0.97, TLI = 0.96, SRMR = 0.08).All BPM subscales were highly correlated with the total score in both caregiver and youth reports (*r* = 0.70–0.80), except caregiver reports for internalizing, which were moderately correlated with total problems (*r* = 0.52). In the BPM subscales, there was a substantial correlation between externalizing and attention problems (caregiver *r* = 0.53, youth *r* = 0.53) but weak correlation between internalizing and externalizing (caregiver *r* = 0.09, youth *r* = 0.33), and internalizing and attention problems (caregiver *r* = 0.03, youth *r* = 0.32).

#### BFS factor structure

We attempted to reproduce Weisz et al.’s (2019) two-factor structure for both the caregiver and youth report versions of BFS through using CFA. Model fit for the two-factor model of the BFS was unsatisfactory (caregiver report: RMSEA = 0.14, CFI = 0.96, TLI = 0.95, SRMR = 0.07, and youth report: RMSEA = 0.10, CFI = 0.97, TLI = 0.96, SRMR = 0.06). As for the BPM, splitting the internalizing factor into a depression factor and an anxiety factor significantly improved model fit for both the caregiver and youth report data. In the BFS Youth, with a 3-factor model, the model fit was acceptable on all the fit indices considered (RMSEA = 0.08, CFI = 0.98, TLI = 0.97, SRMR = 0.05). For the caregiver version, the three-factor model of externalizing, anxiety and depression obtained adequate model fit on three of the four fit indices (RMSEA = 0.11, CFI = 0.98, TLI = 0.97, SRMR = 0.05).

For the caregiver report, three items loaded on a depression factor, with loadings between 0.80 and 0.96, three items loaded on an anxiety factor, with loadings between 0.85 and 0.92, and the remaining six items loaded between 0.81 and 0.90 on an externalizing latent factor. The same items in BFS youth loaded on similar latent factors, with loadings of 0.80– 0.95, 0.73–0.90, and 0.69–0.86, respectively.

Caregiver-reported internalizing and externalizing through the BFS were weakly correlated (*r* = 0.15, *p* < 0.001), but each of the subscales was highly correlated with BFS total problems, with internalizing-total *r* = 0.78, *p* < 0.001 and externalizing-total *r* = 0.73, *p* < 0.001. A similar pattern emerged in the youth-reported BFS, with internalizing-externalizing *r* = 0.22, *p* < 0.001, internalizing-total *r* = 0.87, *p* < 0.001, and externalizing-total *r* = 0.67, *p* < 0.001.

### Convergent validity

Convergent validity analysis was performed by exploring the relationship between BFS and BPM scores. The correlations between the BFS subscales and the BPM subscales are presented in Table 1 (caregiver-report) and Table 2 (youth report).

**Table 1:** Sum score correlations—caregiver report

	BFS int	BFS ext	BPM int	BPM ext	BPM att
BFS int	1.00				
BFS ext	.15**	1.00			
BPM int	.74**	– 016	1.00		
BPM ext	.12*	.83**	.09	1.00	
BPM att	.01	.50**	.03	.53**	1.00

Correlations between measures of similar constructs are in bold

^**^ Significant at the 0.01 level (2-tailed)

^*^ Significant at the 0.05 level (2-tailed)

**Table 2:** Sum score correlations—youth report

	BFS int	BFS ext	BPM int	BPM ext	BPM att
BFS int	1.00				
BFS ext	.22**	1.00			
BPM int	.83**	.19**	1.00		
BPM ext	.17**	.76**	.20**	1.00	
BPM att	.25**	.50**	.33**	.53**	1.00

Correlations between measures of similar constructs in bold

**Significant at the 0.01 level (2-tailed)

The BFS total problem score was highly correlated with BPM total scores for both caregiver (*r* = 0.69, *p* < 0.001) and youth reports (*r* = 0.77 *p* < 0.001). Scores for BFS internalizing were highly correlated with BPM internalizing for both caregiver (*r* = 0.74, *p* < 0.01) and youth reports (*r* = 0.83, *p* < 0.01). Moreover, high correlations were obtained between BFS externalizing and BPM externalizing for caregiver (r = 0.83, *p* < 0.01) and youth reports (*r* = 0.76, *p* < 0.01). Smaller correlations were seen between measures of internalizing and externalizing problems, and moderate associations were obtained between all measures of externalizing problems and BPM attention problems.

### Relations between subscales and diagnostic groups

Overall, 256 participants had one or more internalizing diagnoses and 131 participants had one or more externalizing diagnoses. Fifty-six participants only had diagnoses that we did not code into internalizing or externalizing problems, while 126 were missing diagnoses due to early dropout, early termination of therapy, or administrative errors.

Patients that were given diagnoses with either predominantly internalizing or externalizing difficulties had higher scores on the respective internalizing and externalizing subscales of BFS/BPM. The patients with an internalizing diagnosis reported a 1.9 times higher score on BFS internalizing self-report than the patients without such a diagnosis. Likewise, BFS internalizing caregiver-reported scores were 1.7 times higher for the patients with an internalizing diagnosis. BPM-Y internalizing scores were 1.8 times higher and BPM-P internalizing scores 1.5 times higher for patients with internalizing diagnoses than for those without. For patients with externalizing diagnoses, higher scores were obtained on BFS youth externalizing (1.7 times), BFS caregiver externalizing (1.7 times), BPM-Y externalizing (1.7 times), and BPM-P externalizing (1.5 times) than for those without such diagnoses.

## Discussion

Overall, results showed that the brief measures evaluated in this study, namely the BPM and the BFS, have many qualities that make them ideal for frequent monitoring of youth in therapy. They are eligible for use in measurement-based care by informing practitioners about fluctuations of central dimensions of mental health issues for young patients, prompting therapists to adjust therapy plans.

Weisz et al. [17] found the BFS to be psychometrically sound with a two-factor structure and convergent and discriminant validity in relation to other well-established measures. However, this two-factor structure was not confirmed in our data using a Norwegian population and translation. Rather, we found support for a three-factor model reflective of anxiety, depression, and externalizing problems which had loadings in an expectable range (> 0.69) and a good model fit. Furthermore, indications of validity were also obtained as the subscales correlated with the relevant BPM subscales and were related to relevant diagnoses.

The BPM is the shortened version of the empirically well-established CBCL and YSR. From both US and Norwegian samples, a three-factor model for the BPM has been reported [16] along with indications of convergent validity [13, 15]. A limitation of these studies [13, 15] is that they investigate the BPM in the context of the entire CBCL/YSR and all analyses are based on taking the BPM items out of those measures. These items are then compared with the full scale. This approach, however, could influence the response set and inflate correlations as measurement errors could be overlapping.

In our sample, the three-factor model was not confirmed as a more complex model with four factors (anxiety, depression, externalizing, and attention problems) had a significantly better model fit. However, BPM total scores and subscales were related to the expected BFS subscales and diagnoses, indicating convergent validity. The lack of support for the three-factor model stands in contrast with a recent Norwegian study with a more geographically heterogeneous sample of children from both rural and urban areas [16]. In Pedersen et al. [16] the population is children with elevated internalizing symptoms in the age of 8–12 years, selected for an indicated preventive intervention and hence only provides a test of the parent report (BPM-P). Regarding participants’ age and problem areas, the current study has a broader range with children and youth 6–18 years old referred to treatment for all types of mental health problems. Still, our sample is limited by relying on data from only one facility and with a majority of participants with a high symptom load. Penelo et al. [14] provide support for the expected three-factor model of BPM-P and indications of concurrent validity in a Spanish population. They also present evidence of measurement invariance across age and gender butthe sample was limited to 6 and 8-year-old participants. The measurement invariance across BPM’s entire intended age group should be tested in further research.

For both measures, internal consistency was good to excellent for both subscales and total score in caregiver reports and self-reports. The cross-informant agreement was moderate (from 0.52 to 0.73) between self-reports and caregiver reports. This was expected based on previous studies that often find moderate to weak agreement across different types of informants [18, 19] and illuminates the need for multi-informant approaches in mental health reporting.

Similar constructs measured in the BFS and BPM were highly correlated in all subscales and in both caregiver and youth reports (from 0.74 to 0.83). As expected, moderate correlations were found between attention problems measured by the BPM and externalizing problems in the BFS (caregiver report 0.50, youth report 0.50) and the BPM (caregiver report 0.53 and youth report 0.53). Minor correlations were found across the different constructs of internalizing and externalizing problems.

The expected subscales were elevated among youth with diagnoses of internalizing and externalizing character. In both the BFS and the BPM, substantial and significant differences in scores on the internalizing scale were obtained for those with or without internalizing diagnoses. Similarly, patients with externalizing diagnoses had higher scores on externalizing scales than those without such diagnoses. This is in line with previous findings where higher BPM subscale scores were recorded among participants with theoretically related diagnoses [13]. Larger differences were found in Piper et al. [13], but this was not unexpected as their sample came from a general population. In our clinical sample, we expect more symptoms of internalizing problems also among those with externalizing diagnoses and vice versa. However, both the BFS and the BPM showed the same tendency in our sample.

We tested group differences between gender and found significantly different reporting. In our sample, girls had the highest average internalizing scores on all measures and boys had higher caregiver reported externalizing problems. This is in line with previous knowledge of higher prevalence of anxiety and depression for girls than boys from adolescence and onward [33] and conduct disorders being more frequent in boys than girls [34]. This is also to be expected from data on Norwegian mental health service users where more boys seek help for externalizing problems and more girls seek help for internalizing problems [33, 35].

### Limitations

The current sample was composed entirely of patients who had been referred to the specialist health services and were considered by the treating clinic to fulfill the criteria of at least one ICD diagnosis. Thus, the present study may be influenced by selection factors associated with referrals and evaluations of whether clients should receive treatment. In addition, all participants came from the same clinical facility, hence limiting the generalizability of the finding. Our respondents had, on average, elevated T scores for overall problems in the BPM-Y and BPM-P, in all age groups and both genders. Although BFS and BPM are constructed as clinical tools, it would be of interest to see more evaluations in non-clinical populations or populations with less severe problems.

To get an acceptable model fit, one of the factors was split into two. Splitting the internalizing factor into depression and anxiety significantly increased model fit for both the BFS and the BPM in caregiver and youth reports. Although anxiety and depression are both part of the internalizing problems construct reproduced in numerous studies, a measurement of very few items might have difficulties finding the overlapping aspects between them. Another possibility is that this is an artifact of translation.

The validity checks are limited to correlations between the investigated measures and between the measures and groups of diagnoses. Diagnoses are not distinct indicators of internalizing and externalizing problem factors as there are no theoretical implications in the ICD. Nor can we provide reliability scores for diagnoses as the data rely on a naturalistic diagnostic procedure. Alternatively, diagnoses could have been mapped more systematically, and we suspect that our method of extracting diagnoses from case records is likely to underestimate comorbidity. Another limitation concerning the interpretation of the link between diagnoses and scores on the BFS/BPM is that therapists were not blinded to the results of the BFS/BPM when making diagnoses.

## Conclusion

The BFS and the BPM are short, easy to use, and have acceptable psychometrics for most purposes. Both measure general dimensions in psychopathology and can be useful in both MFS and research with repeated measures. They are easily interpreted, with scores representing total problems and well-known and thoroughly documented factors in youth psychopathology. The parallel forms allow for patient and caregiver perspectives and hence triangulation and a better understanding of cases. BFS is also free to use which makes it easily accessible.

## Data Availability

The dataset supporting the conclusions of this article is not deposited publicly as the data is controlled by the cooperating clinic and not the research group. Data may be available upon request. Contact corresponding author Kristian Rognstad (kristian.rognstad@r-bup.no).

## Appendix 1 Descriptive statistics for BPM-Y, BPM-P, BFS Youth and BFS Caregiver

**Table:**

	Girls	Boys	All participants
BPM-Y Internalizing (n = 393)	6.1 (3.5)	3.8 (3.2)	5.1 (3.5)
BPM-Y Externalizing (n = 393)	3.1 (2.3)	3.4 (2.7)	3.2 (2.5)
BPM-Y Attention (n = 393)	4.4 (2.8)	4.5 (2.9)	4.5 (2.9)
BPM-Y Total (n = 393)	13.6 (6.5)	11.8 (6.8)	12.8 (6.7)
BPM-P Internalizing (n = 415)	6.3 (3.1)	5.5 (3.1)	5.9 (3.1)
BPM-P Externalizing (n = 415)	3.3 (2.9)	4.8 (3.5)	4.0 (3.3)
BPM-P Attention (n = 415)	3.8 (2.9)	5.7 (3.3)	4.7 (3.2)
BPM-P Total (n = 415)	13.5 (6.4)	16.0 (6.8)	14.6 (6.7)
BFS Youth—Internalizing (n = 468)	11.8 (6.4)	7.2 (6.4)	9.8 (6.8)
BFS Youth—Externalizing (n = 468)	4.4 (4.2)	5.1 (4.8)	4.7 (4.5)
BFS Youth—Total (n = 468)	16.2 (8.3)	12.3 (9.1)	14.5 (8.9)
BFS Caregiver—Internalizing (n = 471)	11.3 (6.3)	9.0 (6.5)	10.3 (6.4)
BFS Caregiver—Externalizing (n = 471)	5.7 (5.3)	7.6 (6.3)	6.6 (5.9)
BFS Caregiver—Total (n = 471)	17.1 (9.0)	16.6 (9.8)	16.9 (9.4)

*Note.* Mean scores on scales. Standard deviation in parenthesis

Possible maximum scores for scale:

BPM-Y/BPM-P total = 38, internalizing = 12, externalizing = 14, attention = 12

BFS youth/caregiver total = 48, internalizing = 24, externalizing = 24

## Appendix 2 List of ICD codes dummy coded as internalizing and externalizing diagnoses

Internalizing diagnoses.

- F30-F39
- F40-F48
- F50-F59
- F92-F93
- F940-F942

Externalizing diagnoses.

- F50-F59
- F90-F92
- F941-F942
